# Clinical Characteristics and Aetiology of Uveitis in a Viral Haemorrhagic Fever Zone

**DOI:** 10.21203/rs.3.rs-3222203/v1

**Published:** 2023-08-08

**Authors:** Shiama Balendra, Lloyd Williams, Jalikatu Mustapha, Zikan Koroma, Alicious Kamara, Osman Conteh, Theophilus Kanu, Bangi Saradugu, Santigie Kamara, Laura Ward, Huachun Wang, Tolulope Fashina, Sheku Koroma, Jessica Shantha, Steven Yeh, Alasdair Kennedy

**Affiliations:** King’s Global Health Partnerships; NHS; Royal Free Hospital

## Abstract

**Background/Objectives:**

Studies on uveitis in Sierra Leone were conducted prior to the Ebola Virus Disease epidemic of 2013-16, which was associated with uveitis in 20% of survivors. They did not include imaging or investigation of tuberculosis and used laboratory services outside the country. We performed a cross-sectional study on patients presenting with uveitis to establish their clinical characteristics and identify the impact of in-country laboratory diagnoses.

**Methods:**

We invited uveitis cases presenting to Eye Clinics in Sierra Leone from March to September 2022 to participate in the study. They underwent a diagnostic work-up, including fundus and ocular coherence tomography imaging. Active uveitis cases underwent further investigations including serology and immunological tests for syphilis, tuberculosis and herpetic viruses and HIV, and chest radiographs.

**Results:**

We recruited 128 patients. The mean age was 36 ± 14 years and there was an equal gender split. Panuveitis was the predominant anatomical uveitis type (n=51, 40%), followed by posterior uveitis (n=36, 28%). Bilateral disease affected 40 patients (31%). Active uveitis was identified in 75 (59%) cases. ICD 11 definition of blindness with VA<3/60 occurred in 55 (33%) uveitis eyes. Aetiology of uveitis from clinical and laboratory assessment demonstrated that most cases were of undifferentiated aetiology (n=66, 52%), followed by toxoplasmosis (n=46, 36%). Trauma contributed to eight (6%) cases, syphilis to 5 (4%) cases and Ebola to 2 (2%).

**Conclusions:**

Uveitis was associated with high levels of visual impairment. Posterior and panuveitis contributed to the highest proportion of uveitis cases. Laboratory studies helped differentiate syphilis as a significant aetiology of uveitis.

## Introduction

Uveitis is intraocular inflammation originating from the uveal tract and adjacent structures. Its prevalence varies globally. In the United States of America (USA), a prevalence of 115.3 cases per 100,000 of population has been reported.(1) In India, this was estimated at 714 per 100, 000.(2) Aetiology of uveitis also varies around the world. In the United Kingdom (UK), Fuchs heterochromic uveitis (11.5%), sarcoid (9.7%), idiopathic uveitis (14.9%) and toxoplasmosis (6.9%) were the most common known diagnoses.(3) In the USA these were idiopathic uveitis (34.9%), seronegative spondyloarthropathies (10.4%) and sarcoidosis (9.6%).(4) In China, idiopathic anterior uveitis accounted for 27% of anterior uveitis, and Behcet disease (6.5%) and Vogt-Koyanagi-Harada syndrome (15%) represented the most panuveitis cases.(5) In India, uveitis was caused by tuberculosis in 14.5%, toxoplasmosis in 11.7% and serpiginous choroidopathy in 14.6%.(6)

West Africa has unique epidemiological characteristics with many endemic infectious diseases. It is therefore challenging to extrapolate study results from other parts of the world to make inferences for Sierra Leone's uveitis population. Studies of uveitis aetiology in West Africa are limited. In Nigeria, anatomical subtypes of uveitis patients have been reported and Ayanru reported that the majority of posterior uveitis was of toxoplasmic origin.(7) The role of non-infectious autoimmune-disease related uveitis in Nigeria has also been highlighted.(8) In Benin, 85.7% of 489 patients with uveitis were reported to be idiopathic.(9) The challenges for studies in West Africa to obtain accurate epidemiological data include a lack of retinal imaging equipment and laboratory investigations.

What is known for certain is that uveitis is a significant cause of ocular morbidity in Sierra Leone. In 1992, Ronday published a hospital-based retrospective study. Uveitis was the second leading cause of blindness.(10) In another study by Ronday in 1996, infection accounted for over 50% of cases of uveitis with Toxoplasma Gondii and Treponema Pallidum, causing 43% and 20% of infective cases, respectively.(11) Sierra Leone has a population of 8.3 million and based on the afore mentioned prevalence studies from the USA and India, could have between 9000 and 55,000 cases of uveitis.(1, 12, 13) In reality, this figure may be even higher considering the significant amount of endemic infectious diseases in-country. This represents a significant burden from a condition which affects people throughout their life course. Sight threatening complications such as band keratopathy, cataract, macula oedema and glaucoma, have a major impact on quality of life and blindness also has socioeconomic costs.(14)

Between 2013 and 2016, the Ebola Virus Disease (EVD) epidemic of West Africa affected 28,600 individuals.(15) The PREVAIL study in Liberia reported that 26% of Ebola Virus Disease (EVD) survivors and 12% of control patients show evidence of uveitis, a staggering proportion of individuals with no direct exposure to Ebola infection.(16) Uveitis occurs in approximately 20% of EVD survivors, raising questions regarding its impact on the aetiologic landscape of uveitis.(17)

Although uveitis prevalence data is not available in Sierra Leone, data on other endemic infectious diseases, which may be associated with uveitis and have been reported by the Institute for Health Metrics and Evaluation as part of The Global Burden of Disease Study, may shed light on the scale of the problem.(18) In 2017, the prevalence of tuberculosis was 29%, syphilis was 1% and onchocerciasis (‘River Blindness’) was 5%. There was no estimation for toxoplasmosis or other viruses capable of causing uveitis. HIV, associated with increased risk of toxoplasma or cytomegaloviral uveitis, was confirmed in 17.8%.(19) Sarcoidosis was reported in 0.03%. This contrasts with the USA and the UK which, in 2017, had prevalence rates of 7% and 10% for TB, 0.2% and 0.1% for syphilis, 0.2% for sarcoidosis, 14% and 23% for HIV and 0% rates of Onchocerciasis respectively.(18, 19) The value of diagnostic tests vary according the prevalence of a disease in a specific population.(20)

The aim of this case series is to reveal the pattern of clinical phenotypes, sequelae, and disease associations of uveitis amongst patients presenting to the Eye Departments in Freetown, Sierra Leone. This study presents an opportunity to better understand uveitis in an understudied West African population who have been exposed to recent emergent public health threats including Ebola and Lassa fever. The impact on the population of these viral haemorrhagic fevers associated with uveitis is explored. Given the large number of uveitis sufferers in the country and the low number of ophthalmic trained staff available to manage them with limited resources, this condition represents a significant public health concern.

## Methods

### Study Type and Patient Recruitment

A prospective cross-sectional study was conducted between March to September 2022. With coordination from the National Eye Health Program of the Ministry of Health and Sanitation, patients were recruited from three study sites: Jui Hospital, Lowell and Ruth Gess Eye Hospital and Connaught Government Hospital. Patients identified to have uveitis on assessment were invited to attend a study day at a research eye clinic in Connaught Government Hospital, where a full ophthalmic assessment, fundus photography, ocular coherence tomography were performed and blood tests and chest radiographs were arranged as needed.

### Inclusion and Exclusion Criteria

All patients presenting with active or inactive uveitis of any aetiology were invited to participate in the study. Patients were excluded from the study if they were unable to undergo investigations or if they had active viral haemorrhagic fever, such as EVD or Lassa fever.

### Ethical Considerations

Study protocols were reviewed by the Institutional Review Board and Ethics Committee of Ministry of Health and Sanitation, Sierra Leone and Kings College London and adhere to the tenets of the Declaration of Helsinki. Patients who qualified for protocol evaluation were counselled and consented with the assistance of an interpreter in the patient’s Sierra Leone language or dialect (e.g. Krio, Mende, Temne, and others). Patients who were identified to have uveitis were managed according to the medical judgement of the examining physician in partnership with the eye care providers at the specified sites.

### Investigations

Serology testing was conducted in the laboratory of Connaught Government Hospital after training of laboratory personnel and set up of required equipment and reagents, which had been procured and transported from the UK. Validity and quality control of tests were executed prior to commencement of the study. Patients in the study assessed to have active uveitis had a panel of blood tests performed and a chest radiograph performed. Blood tests included full blood count, ESR, CRP, syphilis (TPHA), ELISA testing for antibodies (VZV IgM, HSV IgG) and TB QuantiFERON and a HIV test if there were clinical suspicion or if systemic steroids were being started. Due to limited reagents, only those with suspected clinical suspicion from history and ocular examination of TB, syphilis, VZV or HSV were offered relevant tests. Other immunological reagents had been procured, including VDRL, VZV IgG, CMV IgG, HSV 1&2 IgG & IgM, Toxoplasma IgM & IgG, however these failed initial validity testing and therefore were not used.

### Data collection

Data were collected by a team of trained research staff and inputted into an anonymized electronic database. The clinical assessment was performed by ophthalmologists and ophthalmic imaging by a team of trained technicians. Laboratory investigations were performed by trained laboratory technicians. Demographic data including name, age, gender, ethnicity, occupation, medical and ocular history were recorded in the study questionnaire. A general ophthalmic examination included visual acuity testing with a Snellen chart, intraocular pressure measurements and a slit lamp examination including a dilated posterior segment assessment. Cases were classified according to the SUN classification: anatomical type, onset/duration/course, anterior segment flare and cells, vitreous haze and activity. (21, 22) Final diagnosis was reached through clinical exam, investigations, and clinical impression.

### Retinal imaging

B-scan ultrasonography was performed for patients without a view of the posterior pole due to media opacity (e.g. cataract, posterior synechiae, vitreous opacity) to assess for vitreous opacity, retinal detachment or posterior segment pathology. Retinal imaging was performed by a trained technician. Imaging equipment used was the Zeiss Clarus 700. Macula centred and mid peripheral images and the Zeiss Cirrus 5000 angioplex. Macula, disc and retinal nerve fibre layers images.

### Outcomes and Statistical Analysis:

SPSS and Microsoft Excel statistical software tools were used. Univariate and multivariate analyses were performed to determine whether demographic variables, clinical presentation (i.e. symptom duration, severity), and anatomic location, and other variables of interest portend a better or poorer visual acuity at presentation and final follow-up. P-value < 0.05 will be considered statistically significant for all analyses.

## Results

### General Characteristics of Uveitis Patients

132 patients were recruited, 128 of these patients were included as part of the study. 3 patients were excluded due to missing data and 1 was excluded as they had been misdiagnosed as uveitis, instead had a pigmentary retinopathy.

The clinical characteristics of the 128 study patients are shown in Table 1. The mean age was 36 (SD ± 14 years and range 5 to 74 years). There were 9 children under the age of 18 years old. Males and female study patients occurred in equal numbers. Patients were predominantly from the Western Urban Area (26.6%, n = 30), which comprises the capital Freetown, and originated mainly from the Temene (25.6%), Mende (18.8%) and Limba (18.0%) ethnic groups. Most patients originated from rural areas of Sierra Leone (53%), rather than urban (43%). Patients reported their highest educational attainment as tertiary or university level (41%), secondary school (36%) and primary school (21%).

### History of Presenting Complaint

Eye pain (68%) and eye redness (67%) were the two most common presenting complaints for seeking medical attention. In 30% of patients, the symptoms had been present for over 3 months. The median duration was 30 days and with a range between 1 day to 20 years. Known risk factors for uveitis on history included 9 (7%) HIV positive patients and 2 (2%) Ebola survivors. Forty-eight (38%) patients had a history of associated trauma, although for most of these cases trauma did not appear to be the underlying aetiology of uveitis.

### Uveitis Classification

Active uveitis was identified in 75 (59%) of cases. Ocular involvement was unilateral in 88 (68.8%) and bilateral in 40 patients (31.2%), with a total of 168 affected eyes in the study. The most common anatomical type of uveitis was panuveitis (40%), followed by posterior uveitis (28%), anterior uveitis (19%) and intermediate uveitis (6%).

### Ocular Findings

Uveitis-associated complications seen in affected eyes included retinal detachment, glaucoma and cataract. Nineteen patients had unilateral or bilateral retinal detachment (14.8%), 34 had glaucoma (26.6%) and 51 had cataract (39.8%).

We assessed sequelae of uveitis and associated visual impairment. We found that uveitis eyes with retinal detachment had worse vision than those without (adjusted means 1.97 vs 0.70, p-value < 0.0001). Those with cataract had worse vision than those without (adjusted means 1.29 vs 0.70, p < 0.01). Those with cataract and retinal detachment compared to those without (adjusted means 1.44 vs 0.45, p = < 0.00001 had significantly worse vision.

### Aetiology

From clinical examination alone, most cases were of undifferentiated aetiology (n = 66, 52%), followed by toxoplasmosis (n = 47, 37%). Trauma was the aetiology of uveitis in 7 patients (5.5%) and syphilis contributed to uveitis in 5 cases (4%). From history and ophthalmic assessment, one patient was considered to have Ebola-related uveitis and another may have been Ebola-related or toxoplasmosis given the clinical appearance. Therefore up to 2% of cases were considered to be caused by Ebola-related uveitis.

107 (83.6%) patients had at least one of the immunological laboratory tests. Of the 32 patients tested, 32% were positive for Quantiferon TB, and 61 patients were positive for HSV IgG (88%). TPHA testing revealed 5 cases (5%) of those tested were positive. VZV IgM was positive in 5 cases (6%) of those tested.

### Imaging

**Figure 1.**
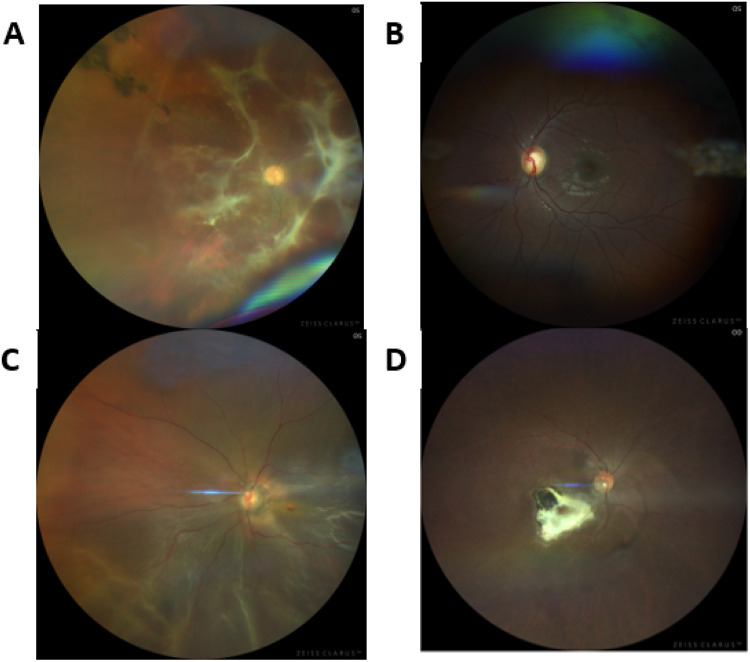
Fundus images were taken with Zeiss Clarus 700 demonstrated ocular sequelae of uveitis. [A] Right fundus photo of chronic vitritis retinal traction [B] Left fundus photograph with evidence of optic disc cupping, a complication of chronic uveitis. [C] Right fundus photo with inferior retinal detachment and round hole. [D] Right fundus photograph with inactive macula chorioretinal scar.

Fundus photography and OCT assessment helped with clinical assessment of uveitis (Fig. 1)

### Level of Blindness

ICD 11 definition of blindness with VA < 3/60 occurred in 33% (n = 55) of uveitis affected eyes. The adjusted mean logMar visual acuity of all uveitis affected eyes (n = 168) was 0.79, (Snellen equivalent 6/38), and of the uveitis affected eyes and worse affected eyes only in bilateral disease (n = 128) the adjusted logMar VA was 1.14, (Snellen equivalent 6/90). Anterior uveitis eyes had significantly better visual acuity than other types, including intermediate, posterior and panuveitis (means of 6/30 vs 6/110, p = 0.013).

## Discussion

This is the first study on uveitis in Sierra Leone to explore its epidemiology and aetiology in since the EVD epidemic of 2013-16. The primary discoveries are that approximately third of individuals with uveitis exhibit bilateral disease and almost 70% have posterior or pan-uveitis. Posterior and panuveitis are associated with significantly worse visual acuity than other types of uveitis. Therefore, similar to the Ronday study over 30 years ago, uveitis remains associated with significant visual impairment and blindness.

A marker of the severity of uveitis seen is represented in the high prevalence of associated ocular complications including retinal detachment, cataract, and glaucoma. The visual impairment associated with ocular sequelae of uveitis demonstrates the burden of the disease within Sierra Leone. The high level of visual impairment related to uveitis-associated retinal detachments and cataracts compared to even glaucoma, which has been widely reported as causing a significant burden of disease in African countries, demonstrates the need for improved management of uveitis and surgical services to manage these conditions.

Within the cohort of patients examined, 2% were found to be associated with Ebola-related uveitis. This suggests that there could potentially be a range of 180 to 11,000 cases of uveitis attributed to Ebola based on estimates of Ebola survivors in Sierra Leone. It's important to note that these figures are derived from reported cases and the actual number of individuals affected may be higher. Inferences on aetiology and therefore investigation and treatment of these patients cannot confidently be made based on other countries’ published data.

It is clear from the history that a lot of patients reported prior associated trauma, although this often was not found to be the aetiology of their uveitis. The prevalence of trauma as an aetiology of uveitis varies significantly in the literature, from less than 1–12%. (23, 24) Studies have demonstrated that there are higher incidence rates of traumatic injuries in low to middle income compared to high income countries. (25, 26) There is also however significant under-reporting and information registration of cases in these settings, so rates may be significantly underestimated.

Compared to epidemiological studies on uveitis in the US and US, undifferentiated uveitis and toxoplasmic uveitis make up the largest proportions of uveitis in Sierra Leone. However, it is likely that in countries with a paucity of resources to confirm aetiologies, this cohort of uveitis patients is over-represented.

Systemic infections causing uveitis, including TB and syphilis are also represented in our cohort. It is likely that in general the prevalence of these systemic infections is underrepresented due to a lack of adequate serological testing. In our cohort, no patients were thought to have uveitis caused by TB as the predominant aetiology, despite 32% of cases tests being QuantiFERON positive. This demonstrates that whilst there is a high prevalence of latent TB in the population generally, few cases within our cohort presented with active TB. Tuberculin skin testing or Mantoux testing combined with intraocular fluid sampling may be more useful given the resource-limited setting. Syphilis testing demonstrated 5% of those tested were positive. All these positive syphilis cases had a change of clinical management based on these results, which demonstrates the value of TPHA / VDRL testing in this cohort of patients with uveitis.

This study represents the first attempt to establish local laboratory services within the main governmental hospital in Freetown, aimed at investigating infectious causes of uveitis. As part of this project, laboratory staff have undergone training on laboratory procedures, handling reagents and equipment, as well as interpreting findings. The utilisation of in-country laboratory facilities presented challenges and limitations, such as power cuts and therefore invalidation of some reagents. However, this project has greater potential of sustainability, increased laboratory expertise and capacity, and the ability to ensure continued provision of necessary supplies for improved management of uveitis patients in the future. Laboratory capacity needs to be built to further characterize infectious and noninfectious causes of uveitis.

The primary findings of this project underscore the continued prevalence of uveitis as a significant cause of visual impairment, particularly in the form of advanced disease as posterior uveitis, panuveitis and bilateral disease. Trauma is also an important risk factor for uveitis and contributed to a significant proportion of disease. The study highlights substantial advancements in the development and application of laboratory investigations for uveitis diagnosis, yet further efforts are required to enhance the differentiation of its underlying causes. Specifically, it is recommended that additional laboratory studies would aid in identifying the aetiology. Obtaining anterior chamber and vitreous samples for processing would be beneficial in obtaining more definitive diagnoses for infectious aetiologies.
